# Switching treatment to cipaglucosidase alfa plus miglustat positively affects patient-reported outcome measures in patients with late-onset Pompe disease

**DOI:** 10.1186/s41687-024-00805-w

**Published:** 2024-11-13

**Authors:** Priya S. Kishnani, Barry J. Byrne, Kristl G. Claeys, Jordi Díaz-Manera, Mazen M. Dimachkie, Hani Kushlaf, Tahseen Mozaffar, Mark Roberts, Benedikt Schoser, Noemi Hummel, Agnieszka Kopiec, Fred Holdbrook, Simon Shohet, Antonio Toscano, on behalf of the PROPEL Study Group, Agnes Sebok, Alan Pestronk, Aleksandra Dominovic-Kovacevic, Aneal Khan, Blaž Koritnik, Celine Tard, Christopher Lindberg, Colin Quinn, Crystal Eldridge, Cynthia Bodkin, David Reyes-Leiva, Derralynn Hughes, Ela Stefanescu, Emmanuelle Salort-Campana, Ernest Butler, Francoise Bouhour, Gee Kim, George Konstantinos Papadimas, Giancarlo Parenti, Halina Bartosik-Psujek, Hashiguchi Akihiro, Heather Lau, Helio Pedro, Henning Andersen, Hernan Amartino, Hideaki Shiraishi, Hiroshi Kobayashi, Ivaylo Tarnev, Jaime Vengoechea, Jennifer Avelar, Jin-Hong Shin, Jonathan Cauci, Jorge Alonso-Pérez, Jozsef Janszky, Julie Berthy, Cornelia Kornblum, Kristina Gutschmidt, Maria Judit Molnar, Marie Wencel, Mark Tarnopolsky, Michel Tchan, Miriam Freimer, Nicola Longo, Nuria Vidal-Fernandez, Olimpia Musumeci, Ozlem Goker-Alpan, Patrick Deegan, Paula R Clemens, Richard Roxburgh, Robert Henderson, Robert Hopkin, Sabrina Sacconi, Simona Fecarotta, Shahram Attarian, Stephan Wenninger, Stephanie Dearmey, Tarekegn Hiwot, Thomas Burrow, Tobias Ruck, Tomo Sawada, Vescei Laszlo, Wolfgang Löscher, Yin-Hsiu Chien

**Affiliations:** 1 Duke University Medical Center, Durham, NC USA; 2 University of Florida, Gainesville, FL USA; 3 Department of Neurology, University Hospitals Leuven, Leuven, Belgium; 4 Laboratory for Muscle Diseases and Neuropathies, Department of Neurosciences, KU Leuven, Leuven, Belgium; 5 John Walton Muscular Dystrophy Research Centre, Newcastle University, Newcastle upon Tyne, UK; 6 Neuromuscular Disorders Unit, Neurology Department, Hospital de la Santa Creu I Sant Pau, Barcelona, Spain; 7 Centro de Investigación en Red en Enfermedades Raras (CIBERER), Barcelona, Spain; 8 Department of Neurology, University of Kansas Medical Center, Kansas City, KS USA; 9 Department of Neurology & Rehabilitation Medicine, University of Cincinnati College of Medicine, Cincinnati, OH USA; 10 Department of Neurology, University of California, Irvine, CA USA; 11 Salford Royal NHS Foundation Trust, Salford, UK; 12 Friedrich-Baur-Institute at the Department of Neurology, LMU University Hospital, LMU Munich, Munich, Germany; 13 Certara GmbH, Lörrach, Germany; 14 Certara, Krakow, Poland; 15 Amicus Therapeutics, Inc., Princeton, NJ USA; 16 Amicus Therapeutics Ltd, Marlow, UK; 17 ERN-NMD Center for Neuromuscular Disorders of Messina, Department of Clinical and Experimental Medicine, University of Messina, Messina, Italy

**Keywords:** Pompe disease, Patient-reported outcomes, Patient-reported Outcome Measurement Information System, Health-related quality of life

## Abstract

**Background:**

Late-onset Pompe disease (LOPD), a rare autosomal recessive multisystemic disorder, substantially impacts patients’ day-to-day activities, outcomes, and health-related quality of life (HRQoL). The PROPEL trial compared cipaglucosidase alfa plus miglustat (cipa+mig) with alglucosidase alfa plus placebo (alg+pbo) in adult patients with LOPD over 52 weeks and showed improved motor and respiratory function in patients switching treatment from standard-of-care enzyme replacement therapy (ERT) to cipa+mig at baseline. This study evaluated the impact of cipa+mig on patient-reported outcomes (PROs), including HRQoL in ERT-experienced patients, using data from PROPEL.

**Methods:**

PROs evaluated included the Subject’s Global Impression of Change (SGIC), Patient-Reported Outcomes Measurement Information System (PROMIS) Physical Function Short Form 20a, PROMIS Fatigue Short Form 8a, Rasch-built Pompe-specific Activity (R-PAct), and European Quality of Life-5 Dimensions 5 Response Levels (EQ-5D-5L). The proportions of responders in the cipa+mig arm and the alg+pbo arm were compared via chi-squared or Fisher’s exact test (patient-level responder analysis), and least squares (LS) mean differences were calculated for change from baseline at Week 52 of the PRO measures (group-level analysis).

**Results:**

At Week 52, patient-level SGIC responder and group-level SGIC analyses favored cipa+mig compared with alg+pbo across all SGIC domains (e.g. 90 vs. 59% responders in the cipa+mig vs. the alg+pbo group for SGIC ability to move around; *P* = 0.0005; and LS mean difference 0.385; *P* = 0.02). Similarly, PROMIS Physical Function and Fatigue domains numerically favored cipa+mig in both analyses (e.g. 50 vs. 40% responders in the cipa+mig vs. alg+pbo arm for PROMIS Physical Function; *P* = 0.37; and LS mean difference 3.1; *P* = 0.11). R-PAct for both treatment groups was similar in the patient-level responder analysis, but numerically favored alg+pbo in the group-level analysis (35% responders in both arms; *P* = 0.95; and LS mean difference −0.8; *P* = 0.48). Self-care, usual activities, and depression/anxiety domains of EQ-5D-5L numerically favored cipa+mig in both analyses (e.g. 20 vs. 12% responders in the cipa+mig vs. alg+pbo arm for EQ-5D-5L self-care; *P* = 0.54; and LS mean difference −0.108; *P* = 0.52).

**Conclusions:**

Overall, switching treatment from alglucosidase alfa to cipa+mig positively impacted PRO measurements during the double-blind period of PROPEL.

**Trial registration:**

NCT03729362; Registration date: November 1, 2018; https://clinicaltrials.gov/study/NCT03729362

**Supplementary information:**

The online version contains supplementary material available at 10.1186/s41687-024-00805-w.

## Abstract (plain language summary)

Late-onset Pompe disease (LOPD) is a rare, multisystemic inherited genetic disease that causes glycogen accumulation in muscles and other body organs, leading to muscle weakness and respiratory insufficiency. LOPD significantly impacts patients’ day-to-day life. Enzyme replacement therapies (ERT) have greatly improved the lives of patients with LOPD. The first approved ERT for LOPD was alglucosidase alfa (alg). To evaluate the effects of a new treatment (cipaglucosidase alfa+miglustat [cipa+mig]) in adult patients with LOPD, two-thirds of patients were switched from alg to cipa+mig and the remaining patients continued receiving alg (alg+placebo [alg+pbo]). We used patient-reported outcome (PRO) questionnaires (asking patients how they feel) to assess changes in patient health. Groups were similar at baseline. Analyses showed that patients improved following cipa+mig treatment for all domains of the PROs Subject’s Global Impression of Change (SGIC; overall physical well-being, effort of breathing, muscle strength, muscle function, ability to move around, activities of daily living, energy level, level of muscular pain) and the Patient-Reported Outcomes Measurement Information System (PROMIS; Physical Function, Fatigue) compared with when treated with alg+pbo. Rasch-built Pompe-specific Activity (R-PAct), a survey evaluating daily activities and social life of patients living with Pompe disease, showed that patients felt similar after cipa+mig and alg+pbo. European Quality of Life-5 Dimensions-5 Response Levels (EQ-5D-5L), a measure of health covering five dimensions, favored cipa+mig in the self-care, usual activities, pain/discomfort and depression/anxiety areas, and alg+pbo for mobility. Overall, changing treatment from alg to cipa+mig positively affects PROs and the patient’s general well-being.

**Supplementary information:**

The online version contains supplementary material available at 10.1186/s41687-024-00805-w.

## Background

Pompe disease, a rare autosomal recessive multisystemic disorder caused by the deficiency of the lysosomal glycogen-hydrolyzing enzyme, acid alpha-glucosidase (GAA), causes glycogen buildup in the lysosomes [1–3]. Based on the presence or absence of cardiomyopathy in the first year of life, the disease is broadly classified as severe infantile-onset and chronically progressive late-onset Pompe disease (LOPD), respectively; both forms of the disease can lead to irreversible damage to muscle function [4, 5]. LOPD primarily affects skeletal muscles, leading to progressive muscle weakness, respiratory difficulties, reduced mobility, and fatigue [6, 7].

A cornerstone treatment for LOPD is enzyme replacement therapy (ERT) with a recombinant human GAA (rhGAA), alglucosidase alfa, which has significantly improved the clinical outcomes in patients [8–11]. Although alglucosidase alfa has substantially improved the symptoms and slowed the progression of the disease, studies have shown that the response to alglucosidase alfa plateaus or declines over time, and the disease progresses in a vast majority of patients [12–15]. Consequentially, treatment options with sustained efficacy were required to improve the lives of patients with Pompe disease. Recently, two new treatments have been approved for Pompe disease (avalglucosidase alfa and cipaglucosidase alfa plus miglustat [cipa+mig]), both evaluated in head-to-head trials against alglucosidase alfa [16, 17]. Cipaglucosidase alfa, an rhGAA, administered along with a small-molecule enzyme stabilizer, miglustat, is a novel, two-component therapy for Pompe disease [17, 18]. Cipaglucosidase alfa in combination with miglustat was recently approved in the European Union, United Kingdom, and United States of America for treating adults with LOPD [19–21]. The efficacy and safety of cipa+mig have been evaluated in the phase 3, randomized, double-blind, placebo-controlled, PROPEL trial (NCT03729362) in adult patients with LOPD who were either ERT naïve or had received the standard-of-care ERT, alglucosidase alfa, for ≥2 years (ERT experienced) [17]. The results of the study showed that switching treatment from the standard of care to cipa+mig in ERT-experienced patients statistically favored key endpoints (6-minute walk distance [6MWD] and % predicted forced vital capacity [FVC]), and numerically favoured other secondary outcomes that assessed for motor, pulmonary, and muscle functions, and Patient-reported Outcome Measurement Information System (PROMIS) Physical Function and Fatigue scores, compared with alglucosidase alfa plus placebo (alg+pbo) [17].

Chronic and debilitating conditions such as LOPD significantly impact the day-to-day activities, outcomes, and health-related quality of life (HRQoL) of patients, and the clinical signs and symptoms such as muscle weakness and atrophy, fatigue, and pain affect patients’ physical, emotional, and social well-being [22]. Patients with LOPD require increased medical care and support with daily activities, and many limit or cease their employment [23–26]. Patient-reported outcomes (PRO) measures are important endpoints to assess the impact of disease and therapeutic outcomes on patients’ lives [27, 28]. The Rasch-built Pompe-specific Activity (R-PAct) scale, a PRO instrument designed to evaluate disease progression in LOPD, explicitly assesses the limitations patients face in their daily activities and social participation [29]. Moreover, physical disability and fatigue are among the important symptoms experienced by patients with LOPD [30], and hence, PROMIS tools are also useful in assessing the experiences of patients living with LOPD [22, 31]. The European Quality of Life-5 Dimensions 5 Response Levels (EQ-5D-5L) [32], a multi-attribute health measure less commonly used in LOPD, is a valid and reliable instrument that has been reported to have good psychometric properties [33]. EQ-5D-5L was used to estimate health-state utilities for LOPD in a vignette study in the United Kingdom using the PROPEL trial data [34]. The Subject’s Global Impression of Change (SGIC) evaluates the difference between the patient’s current and previous health state and asks if the overall well-being or a specific health area improved, stayed the same, or worsened. The SGIC has been validated, e.g. in fibromyalgia [35]. Since switching treatment from standard of care to cipa+mig improved motor, respiratory, and muscle function in ERT-experienced patients in the PROPEL trial, we evaluated whether there was an impact of switching treatment on several PRO measures by exploring data from the PROPEL study.

## Methods

This study evaluated the PROs in ERT-experienced patients using data from the PROPEL study. Patients enrolled in the PROPEL study were randomized 2:1 to receive either cipa+mig (*n* = 85) or alg+pbo (*n* = 38) for 52 weeks (double-blind period). They had either never been treated with an ERT before (i.e., ERT naïve) or had received the standard-of-care ERT, 20 mg/kg alglucosidase alfa, once every other week for ≥2 years (i.e., ERT experienced). PROPEL was approved by independent ethics committees and institutional review boards at each study site and was conducted according to international guidelines for clinical studies, such as the Declaration of Helsinki and Good Clinical Practice Guidelines. Additional details of the study protocol have been detailed elsewhere [17].

### Data collection

We analyzed the following PRO measures, which reflect self-reported changes of quality of life; see also Table 1.

(i) The SGIC [17, 35–37] consists of eight items (overall physical well-being, effort of breathing, muscle strength, muscle function, ability to move around, activities of daily living, energy level, and level of muscular pain). Each of the eight items is scored on a 7-point rating scale, with answers as follows: 1 = very much worse; 2 = worse; 3 = somewhat worse; 4 = no change; 5 = somewhat improved; 6 = improved; and 7 = very much improved. For the analysis, patients with SGIC item scores ≥4 at Week 52 were classified as ‘responders’.
(ii) PROMIS Physical Function Short Form 20 (v2.0) [17, 38, 39] consists of 20 questions. The first 14 questions are scored on a scale of 1 to 5 with responses as follows: 1 = unable to do; 2 = with much difficulty; 3 = with some difficulty; 4 = with a little difficulty; and 5 = without any difficulty. The next six questions are scored on a scale of 1 to 5 and have the following responses: 1 = cannot do; 2 = quite a lot; 3 = somewhat; 4 = very little; and 5 = not at all. The total score ranges between 20 and 100, with a higher score indicating better physical functioning. For the analyses, patients with a change from baseline >0 in PROMIS Physical Function scores at Week 52 were classified as ‘responders’.
(iii) PROMIS Fatigue Short Form 8a consists of eight questions, scored on a scale of 1 to 5 as follows: 1 = not at all; 2 = a little bit; 3 = somewhat; 4 = quite a bit; and 5 = very much. Two questions each are scored on a 1 to 5 scale with responses as follows: 1 = never; 2 = rarely; 3 = sometimes; 4 = often; and 5 = always [17]. The total score ranges between 8 and 40, with lower scores indicating less fatigue. Patients with a change from baseline <0 in PROMIS Fatigue scores at Week 52 were classified as ‘responders’ in the analysis.
(iv) R-PAct [29] questionnaire is designed to evaluate the effect of Pompe disease on the patient’s daily activities and social life. It consists of 18 questions scored on a scale from 0 to 2 with 0 = no; 1 = yes, but with difficulty; 2 = yes, without difficulty. The total R-PAct score is based on the summed-up score across 18 items, which ranges from 0 to 36, with higher scores indicating less impact of the disease on the muscles. In the analysis, patients with a change from baseline >0 in R-PAct scores were classed as ‘responders’.
(v) EQ-5D-5L is a health status measure consisting of the EQ-5D descriptive system and the EQ-visual analogue scale (VAS). The EQ-5D descriptive system covers five dimensions (mobility, self-care, usual activities, pain/discomfort, and depression/anxiety) with five categorical responses as follows: Level 1 = indicating no problem; Level 2 = indicating slight problems; Level 3 = indicating moderate problems; Level 4 = indicating severe problems; Level 5 = indicating extreme problems for pain and anxiety or indicating ‘unable to’ for mobility, self-care, and activity [17, 32]. Patients were classified as responders if the change from baseline to Week 52 was <0 for a dimension or if a patient scored 1 at both baseline and Week 52 for a dimension. The EQ-VAS is a quantitative measure of health outcome that reflects the patient’s self-rated health on a vertical VAS from 0 to 100, where the endpoints are labeled ‘The worst health you can imagine’ and ‘The best health you can imagine’, respectively. Patients were classified as responders if the change in EQ-VAS at Week 52 was ≥10%.
(vi) The EQ-5D-5L index value is a single summary number that reflects how good or bad a health state (5-digit code from the EQ-5D descriptive system) is according to the preferences of the general population of a country or region. The index value is calculated by attaching single weights to each of the five levels in each of the five dimensions and subtracting the resulting weight from one, the value for the state of full health (i.e., the state 11111) [40]. Patients were classified as responders if the change in EQ-5D-5L index value was >0.

**Table 1:** Baseline characteristics of ERT-experienced patients treated with cipa+mig or alg+pbo

Parameters	**Cipa+mig** (*n* = 65)	**Alg+pbo** (*n* = 30)
Age (years), median (range)	48.0 (21–74)	46.5 (24–66)
Age at diagnosis (years), median (range)	39.0 (1–63)	39.0 (7–62)
Males, n (%)	28 (43.1)	14 (46.7)
Race, n (%)		
Asian	3 (4.6)	1 (3.3)
Japanese	2 (3.1)	4 (13.3)
American Indian or Alaska Native	0	1 (3.3)
Black or African American	0	1 (3.3)
White	55 (84.6)	22 (73.3)
Other	5 (7.7)	1 (3.3)
ERT duration (years), median (Q1–Q3)	7.6 (4.3–10.2)	7.1 (3.8–10.4)

There were no statistically significant differences in baseline characteristics between the two treatment groups

*Alg+pbo* alglucosidase alfa plus placebo, *cipa+mig* cipaglucosidase alfa plus miglustat, *ERT* enzyme replacement therapy, *n* subset population, *Q* quartile

### Statistical analysis

For all PRO measures, patient-level responder analyses compared the proportion of patients satisfying literature-based responder thresholds in cipa+mig versus alg+pbo groups from baseline to Week 52 using chi-squared or Fisher’s exact tests. The group-level analyses for the PRO outcomes were based on the between-group differences (cipa+mig vs. alg+pbo) for change from baseline to Week 52 using analysis of covariance (ANCOVA) adjusted for the baseline value (as a continuous covariate), as well as baseline age, sex, height, and weight. Nominal p-values were calculated, not adjusting for multiple comparisons.

## Results

### Baseline characteristics

Overall, most patients (77.2%) enrolled in the PROPEL study (*n* = 123) had received ERT with alglucosidase before the study entry [17]. The baseline characteristics of the ERT-experienced patients who received cipa+mig or alg+pbo are shown in Table 1. The median (Q1–Q3) duration of ERT in the cipa+mig group was 7.6 years (4.3–10.2), and that of the alg+pbo group was 7.1 years (3.8–10.4).

### SGIC in ERT-experienced patients after cipa+mig or alg+pbo treatment

**Fig. 1:**
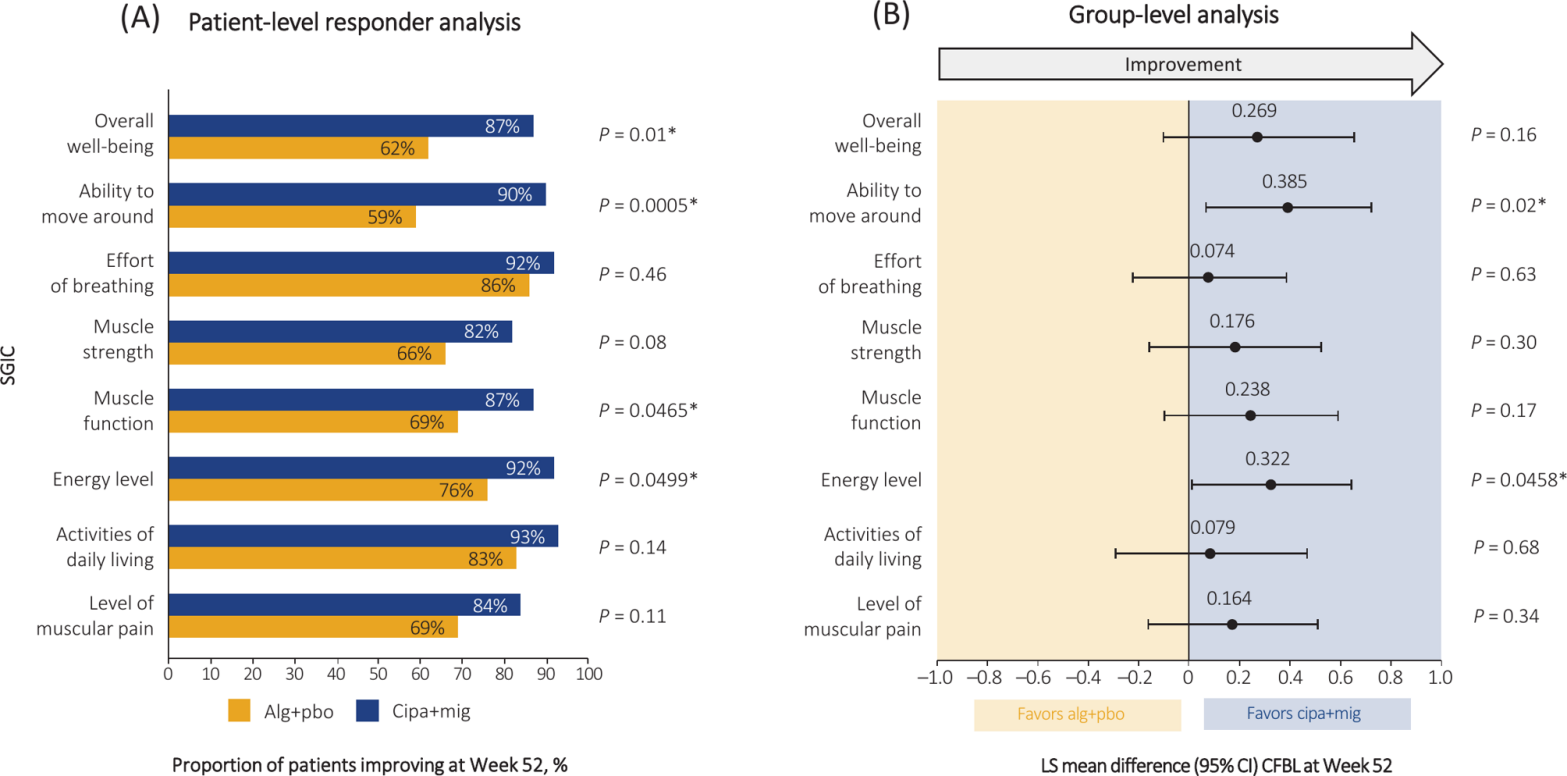
Patient-level responder analysis (**A**) and group-level analysis (**B**) ofSGIC in ERT-experienced patients. Patients with item scores ≥4 at Week 52 were classified as ‘responders’ in the SGIC analysis. *Indicates nominal significance not adjusted for multiplicity. *Alg *alglucosidase alfa; *CFBL *change from baseline; *CI *confidence interval; *cipa *cipaglucosidase alfa; *ERT* enzyme replacement therapy; *LS* least squares; *mig *miglustat; *pbo *placebo; *SGIC *Subject’s Global Impression of Change

The patient-level responder analysis showed that, at Week 52, a higher proportion of the ERT-experienced patients in the cipa+mig group had improved SGIC from baseline across all domains compared with the alg+pbo group (Fig. 1A). A similar trend was observed for the group-level analysis where all SGIC domains favored cipa+mig compared with alg+pbo treatment (Fig. 1B). In the patient-level responder analysis, a nominal statistical significance was achieved across four domains (overall physical well-being [*P* = 0.01], ability to move around [*P* = 0.0005], muscle function [*P* = 0.0465], and energy level [*P* = 0.0499]) and across two domains (ability to move around [*P* = 0.02] and energy level [*P* = 0.0458]) in the group-level analysis.

### Effect of cipa+mig or alg+pbo on PROMIS Physical Function and Fatigue domains, and R-PAct outcomes

**Fig. 2:**
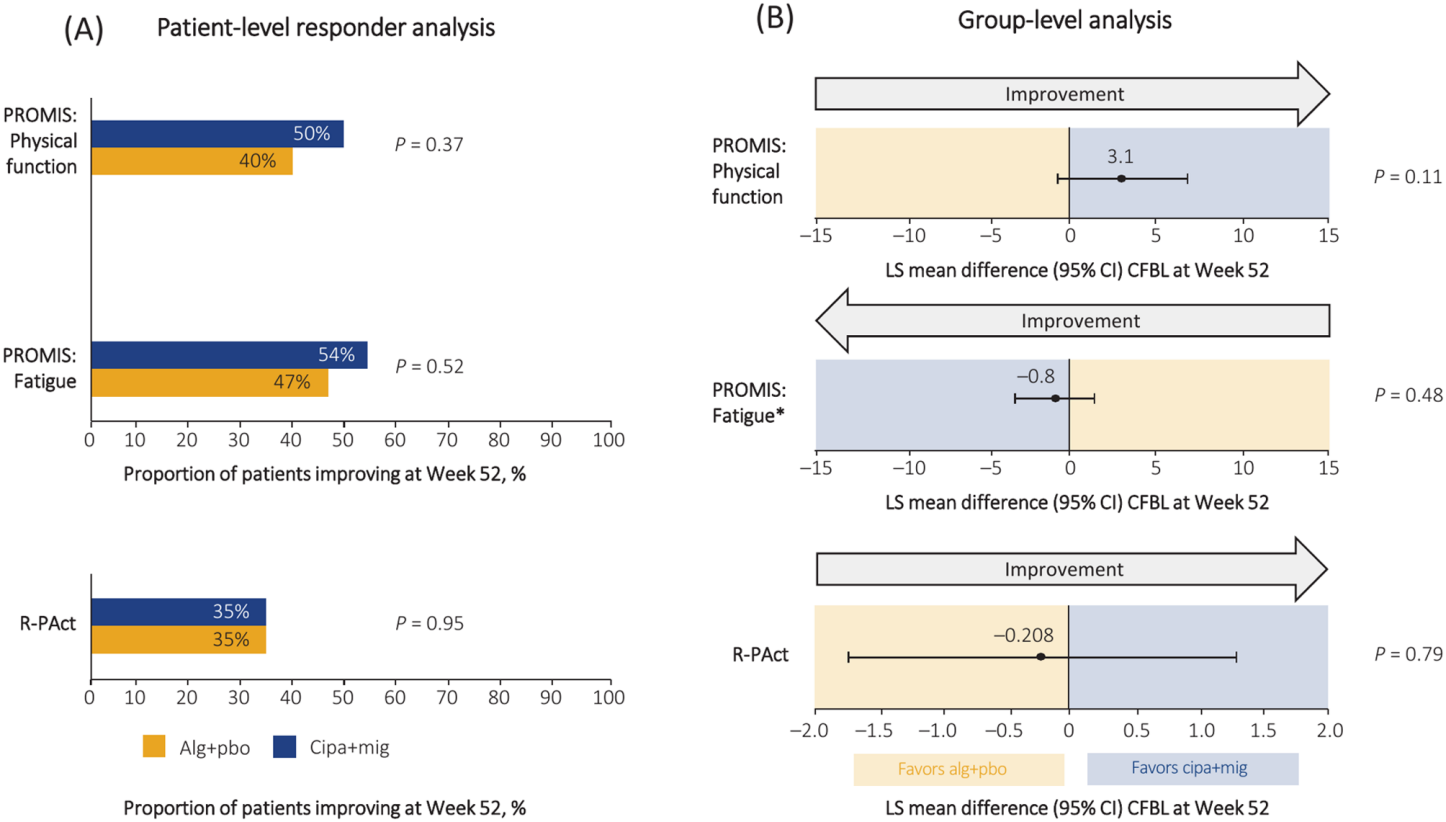
Patient-level responder analysis (**A**) and group-level analysis (**B**) of PROMIS physical and fatigue domains and R-PAct outcomes in ERT-experienced patients. Patients with a change from baseline >0 in PROMIS Physical Function or R-PAct, or <0 for PROMIS Fatigue, were classed as ‘responders.’ *For the Fatigue endpoint, a negative change from the baseline value indicated a better health outcome. *alg* alglucosidase alfa, *CFBL* change from baseline, *CI* confidence interval, *cipa* cipaglucosidase alfa, *ERT* enzyme replacement therapy, *LS* least squares, *mig* miglustat, *pbo* placebo, *PROMIS* Patient-Reported Outcome Measurement Information System, *R-Pact* Rasch-built Pompe-specific Activity

The patient-level responder analysis showed that a higher proportion of the ERT-experienced patients in the cipa+mig group had improved PROMIS Physical Function and Fatigue scores compared with the alg+pbo group (Fig. 2A). Both PROMIS Physical Function and Fatigue domains numerically favored cipa+mig in group-level analyses (Fig. 2B). However, the proportion of ERT-experienced patients with improved R-PAct outcomes was the same for both cipa+mig and alg+pbo (35%) in the patient-level responder analysis (Fig. 2A). The R-PAct outcome was similar for both groups and favored alg+pbo in the group-level analysis (Fig. 2B).

### The impact of cipa+mig or alg+pbo on EQ-5D-5L in ERT-experienced patients

**Fig. 3:**
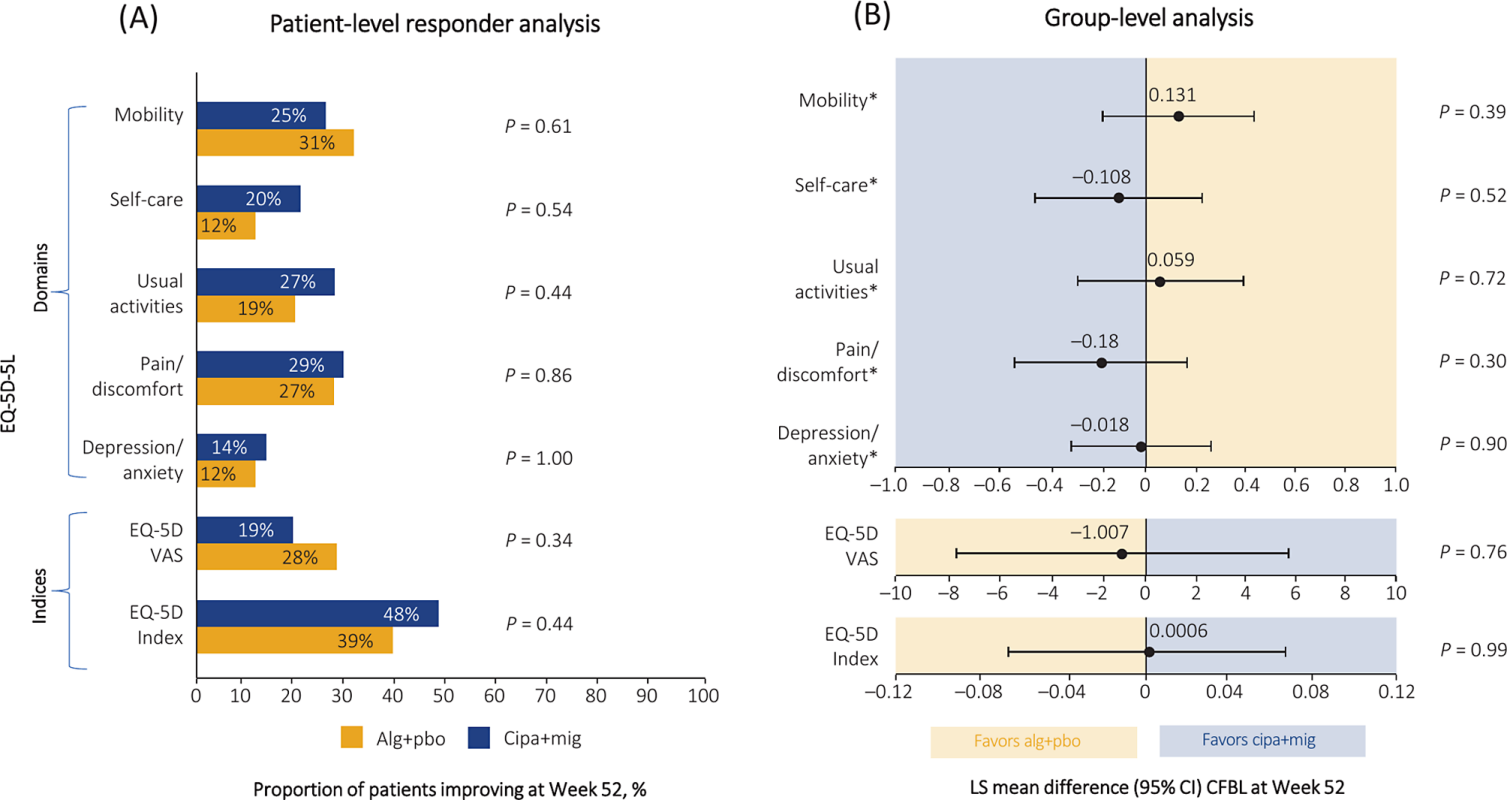
Patient-level responder analysis (**A**) and group-level analysis (**B**) of impact of treatment on EQ-5D-5L in ERT-experienced patients. Patients were classed as responders if the change from baseline to Week 52 was <0 for EQ-5D-5L item scores or if a patient scored 1 at both baseline and Week 52 for an item, if the change in EQ-5D-5L index value was >0, or if the change in EQ-5D-5L VAS at Week 52 was ≥ 10%. *For these endpoints, a negative CFBL value indicated a better result. *Alg* alglucosidase alfa, *CFBL* change from baseline, *CI* confidence interval, *cipa* cipaglucosidase alfa, *ERT* enzyme replacement therapy, *EQ-5D-5L* EuroQol 5 Dimensions-5 Levels instrument, *LS* least squares, *mig* miglustat, *pbo* placebo, *VAS* visual analogue scale

In the EQ-5D-5L descriptive system, four out of five domains (self-care, usual activities, pain/discomfort, and depression/anxiety) favored cipa+mig in the patient-level responder analysis (Fig. 3A). Self-care, pain/discomfort, and depression/anxiety domains favored cipa+mig, whereas mobility and usual activities domains favored alg+pbo in the group-level analyses (Fig. 3B). EQ-5D-5L mobility and EQ-5D-VAS favored alg+pbo for patient-level responder as well as group-level analysis, whereas EQ-5D index value favored cipa+mig in both analyses (Fig. 3A and B).

## Discussion

The results of our study demonstrate that during the double-blind phase of PROPEL, cipa+mig led to an overall improvement of PROs in ERT-experienced patients with LOPD, i.e. in patients switching to cipa+mig at the baseline of PROPEL. All SGIC domains favored cipa+mig vs. alg+pbo for patient-level responder and group-level analyses, with nominal statistical significance for four domains in the patient-level responder analysis, and two domains in the group-level analysis. The SGIC item ‘effort of breathing’ showed a non-significant advantage of cipa+mig over alg+pbo, while FVC results significantly favored cipa+mig in the PROPEL study [17]. Differences between subjective patient-reported symptoms (e.g. breathlessness) and objective measures (e.g. FVC) have been observed in other disease areas such as idiopathic pulmonary fibrosis [41]. These observations suggest that these types of measures capture different information and both should be used in order to provide complementary insights into a treatment’s impact. PROMIS domains numerically favored cipa+mig in both the patient-level responder and group-level analyses. R-PAct outcomes were similar for both cipa+mig and alg+pbo. Four out of five EQ-5D-5L domains favored cipa+mig in the patient-level responder analysis, and measurements were evenly balanced in the group-level analysis.

Alglucosidase alfa, the first available ERT for LOPD for well over a decade, has significantly improved the symptoms and clinical outcomes in patients with LOPD. However, the effect of alglucosidase alfa diminishes within a few years of therapy, resulting in a sustained secondary decline in several clinical outcome measures and impacting the long-term course of the disease [9, 12, 13]. However, switching treatment from alglucosidase alfa to cipa+mig at the baseline of the PROPEL trial showed an improvement in the overall well-being of those ERT-experienced patients as reflected in the PRO measures assessed in this study.

Among the various PRO measures used in this study, PROMIS Physical Function 20a is an important tool for measuring physical function in patients with LOPD and has previously shown validity for use in LOPD [31].

## Limitations of the study

The PROPEL trial was not powered to compare cipa+mig and alg+pbo concerning PROs, and therefore, the analyses presented in this manuscript should be regarded as secondary, exploratory, post hoc analyses. No adjustment for multiple testing was applied due to the exploratory nature of this analysis. However, the advantage of using PROPEL data was that we could evaluate the PROs in a well-sized cohort, considering the rarity of LOPD. Of the PROs evaluated in this study, SGIC has not yet been validated for Pompe disease, but has been used as a measure of patient-relevant changes in other recent Pompe disease clinical trials [16]. Moreover, it was considered a patient-relevant endpoint to assess morbidity in LOPD by the German Federal Joint Committee [42]. Another limitation is the follow-up time of PROPEL of only one year, and future studies are needed to confirm the long-term positive impact of cipa+mig on patient-reported outcomes, including HRQoL.

## Conclusions

These analyses demonstrate that switching treatment from alg+pbo to cipa+mig has a positive impact on PRO measurements, suggesting that cipa+mig is an important new treatment option for managing LOPD. Cipa+mig benefits HRQoL and highlights the importance of capturing the patient’s perspective in LOPD. Continued research will elucidate the long-term and real-world impact of cipa+mig on patients’ quality of life.

## Electronic supplementary material

Below is the link to the electronic supplementary material.

Supplementary Material 1

## Data Availability

Data sharing proposals and requests for data from the PROPEL study will be reviewed on a case-by-case basis. Requests for data should be addressed to Nick Rees at nrees@amicusrx.com. Requests will be reviewed by a medical steering committee.

## Abbreviations

6MWD: 6-minute walk distance
alg+pbo: Alglucosidase alfa plus placebo
ANCOVA: Analysis of covariance
cipa+mig: Cipaglucosidase alfa plus miglustat
EQ-5D-5L: European Quality of Life-5 Dimensions 5 Response Levels
ERT: Enzyme replacement therapy
FVC: Forced vital capacity
GAA: Acid alpha-glucosidase
HRQoL: Health-related quality of life
LOPD: Late-onset Pompe disease
LS: Least squares
PRO: Patient-reported outcome
PROMIS: Patient-Reported Outcomes Measurement Information System
rhGAA: Recombinant human GAA
R-PAct: Rasch-built Pompe-specific Activity
SGIC: Subject’s Global Impression of Change
VAS: Visual analogue scale
