# Supplementary material for: Switching treatment to cipaglucosidase alfa plus miglustat positively affects patient-reported outcome measures in patients with late-onset Pompe disease
Source: J Patient Rep Outcomes. 2024 Nov 13;8:132. doi: 10.1186/s41687-024-00805-w (PMC11561219; doi:10.1186/s41687-024-00805-w)
Supplement: Supplementary file 1 — Supplementary Material 1 [file 41687_2024_805_MOESM1_ESM.pdf]

Supplementary Material for: Switching treatment to cipaglucosidase alfa plus miglustat positively affects patient-reported outcome measures in patients with late-onset Pompe disease

Supplementary Table 1

PRO measure	Description	Validated in Pompe?	Definition of response
SGIC	The SGIC assesses the patients' self-perceived change in their condition over time. It consists of eight items (overall physical well-being, effort of breathing, muscle strength, muscle function, ability to move around, activities of daily living, energy level, and level of muscular pain). Each of the eight items is scored on a 7-point rating scale, with answers as follows: 1 = very much worse; 2 = worse; 3 = somewhat worse; 4 = no change; 5 = somewhat improved; 6 = improved; and 7 = very much improved. Recall period: beginning of the PROPEL trial.	No	Response was defined as no deterioration at Week 52 compared with baseline, i.e., SGIC item score of 4 (no change), 5 (somewhat improved), 6 (improved) or 7 (very much improved) at Week 52.
PROMIS Physical Function Short Form 20 (v2.0)	PROMIS Physical Function Short Form 20 (v2.0) measures self-reported capability of physical activities, including functioning of upper and lower extremities and central regions, and instrumental activities of daily living. It consists of 20 questions. The first 14 questions relate to the ability to perform activities of daily living such as doing chores, dressing up and washing, and are scored on	Yes [1]	Response was defined as at least marginal improvement at Week 52 compared with baseline, i.e., PROMIS Physical Function score change

PRO measure	Description	Validated in Pompe?	Definition of response
	a scale of 1 to 5 with responses as follows: 1 = unable to do; 2 = with much difficulty; 3 = with some difficulty; 4 = with a little difficulty; and 5 = without any difficulty. The next six questions cover limitations due to health in performing physical activities such as running, lifting heavy objects and walking long distances, and are scored on a scale of 1 to 5 and have the following responses: 1 = cannot do; 2 = quite a lot; 3 = somewhat; 4 = very little; and 5 = not at all. The raw total score ranges between 20 and 100. Raw scores are converted to T-scores, with the general population average set at 50 and a standard deviation of 10. Higher T-scores indicate better physical functioning. Recall period: now.		from baseline >0 at Week 52
PROMIS Fatigue Short Form 8a	PROMIS Fatigue Short Form 8a evaluates fatigue in terms of its physical, mental, and social impact. It consists of eight questions, scored on a scale of 1 to 5. Six questions cover, for example, degree of fatigue, impact on physical activities and interference with daily activities, and have responses as follows: 1 = not at all; 2 = a little bit; 3 = somewhat; 4 = quite a bit; and 5 = very much. Two questions cover the frequency of fatigue precluding from	No	Response was defined as at least marginal improvement at Week 52 compared with baseline, i.e., PROMIS Fatigue score change from baseline <0 at Week 52

PRO measure	Description	Validated in Pompe?	Definition of response
	starting and finishing things, and have responses as follows: 1 = never; 2 = rarely; 3 = sometimes; 4 = often; and 5 = always. The raw total score ranges between 8 and 40. Raw scores are converted to T-scores, with the general population average set at 50 and a standard deviation of 10. Higher T-scores indicate greater levels of fatigue. Recall period: past 7 days.		
R-PAct	The R-PAct questionnaire is designed to evaluate the effect of Pompe disease on the patients' daily activities and social life. It consists of 18 questions scored on a scale from 0 to 2 with 0 = no; 1 = yes, but with difficulty; and 2 = yes, without difficulty. The total R-PAct score is based on the summed-up score across 18 items, which ranges from 0 to 36, with higher scores indicating less impact of the disease on the muscles.	Yes [2]	Response was defined as at least marginal improvement at Week 52 compared with baseline, i.e., R-PAct score change from baseline >0 at Week 52
EQ-5D-5L	EQ-5D-5L is a health status measure consisting of the EQ-5D descriptive system and the EQ-VAS. The EQ-5D descriptive system covers five dimensions (mobility, self-care, usual activities, pain/discomfort, and depression/anxiety) with five categorical responses as follows: Level 1 = no problem; Level 2 = slight problems; Level 3 = moderate problems; Level 4 = severe	No	EQ-5D descriptive system: Response was defined as at least marginal improvement at Week 52 compared with baseline, or staying at best health at

PRO measure	Description	Validated in Pompe?	Definition of response
	problems; and Level 5 = ‘extreme problems’ for pain and anxiety or ‘unable to’ for mobility, self-care, and activity, i.e., higher levels correspond to worse health. The EQ-VAS is a quantitative measure of health outcome that reflects the patient’s self-rated health on a vertical VAS from 0 to 100, where the endpoints are labelled ‘The worst health you can imagine’ and ‘The best health you can imagine’, respectively. The EQ-5D-5L index value is a single summary number that reflects how good or bad a health state (5-digit code from the EQ-5D descriptive system) is according to the preferences of the general population of a country or region. The index value is calculated by attaching single weights to each of the 5 levels in each of the 5 dimensions and subtracting the resulting weight from 1, the value for the state of full health (i.e., the state 11111). Higher EQ-5D-5L index values indicate better health.		Week 52, i.e., change from baseline <0 at Week 52 for a dimension, or a patient scored 1 at both baseline and Week 52 for a dimension [3]<br><br>EQ-VAS: Response was defined as at least 10% improvement at Week 52 compared with baseline, i.e., percentage change from baseline ≥10% at Week 52<br><br>EQ-5D-5L index value: Response was defined as at least marginal improvement at Week 52

PRO measure	Description	Validated in Pompe?	Definition of response
			compared with baseline, i.e., change from baseline >0 at Week 52

EQ-5D-5L: European Quality of Life-5 Dimensions 5 Response Levels; PRO, patient-reported outcome; PROMIS: Patient-Reported Outcomes Measurement Information System; R-PAct: Rasch-built Pompe-specific activity; SGIC: Subject's Global Impression of Change; VAS: Visual Analogue Scale

Supplemental References

[1] Kishnani PS, Shohet S, Raza S, Hummel N, Castelli JP, Sitaraman Das S et al (2024) Validation of the Patient-Reported Outcomes Measurement Information System (PROMIS®) physical function questionnaire in late-onset Pompe disease using PROPEL phase 3 data. J Patient Rep Outcomes 8(1):13.

[2] van der Beek NA, Hagemans ML, van der Ploeg AT, van Doorn PA, Merkies IS (2013) The Rasch-built Pompe-specific activity (R-PAct) scale. Neuromuscul Disord 23(3):256-264.

[3] Toscano A, Pollissard L, Msihid J, van der Beek N, Kishnani PS, Dimachkie MM et al. (2024) Effect of avalglucosidase alfa on disease-specific and general patient-reported outcomes in treatment-naïve adults with late-onset Pompe disease compared with alglucosidase alfa: Meaningful change analyses from the Phase 3 COMET trial. Mol Genet Metab 141(2):108121.
